# Design and Simulation Investigation of Si_3_N_4_ Photonics Circuits for Wideband On-Chip Optical Gas Sensing around 2 µm Optical Wavelength

**DOI:** 10.3390/s21072513

**Published:** 2021-04-03

**Authors:** Natnicha Koompai, Papichaya Chaisakul, Pichet Limsuwan, Xavier Le Roux, Laurent Vivien, Delphine Marris-Morini

**Affiliations:** 1 Centre de Nanosciences et de Nanotechnologies, Université Paris Sud, CNRS, Université Paris Saclay, 91120 Palaiseau, France; natnicha.koompai@u-psud.fr (N.K.); xavier.leroux@u-psud.fr (X.L.R.); laurent.vivien@c2n.upsaclay.fr (L.V.); delphine.morini@u-psud.fr (D.M.-M.); 2 Department of Physics, Faculty of Science, Kasetsart University, Bangkok 10900, Thailand; 3 Department of Physics, Faculty of Science, King Mongkut’s Institute of Technology Ladkrabang, Bangkok 10520, Thailand; pichet.li@kmitl.ac.th

**Keywords:** Si_3_N_4_ on SiO_2_, multi-mode interferometer, short-wave infrared

## Abstract

We theoretically explore the potential of Si_3_N_4_ on SiO_2_ waveguide platform toward a wideband spectroscopic detection around the optical wavelength of 2 μm. The design of Si_3_N_4_ on SiO_2_ waveguide architectures consisting of a Si_3_N_4_ slot waveguide for a wideband on-chip spectroscopic sensing around 2 μm, and a Si_3_N_4_ multi-mode interferometer (MMI)-based coupler for light coupling from classical strip waveguide into the identified Si_3_N_4_ slot waveguides over a wide spectral range are investigated. We found that a Si_3_N_4_ on SiO_2_ slot waveguide structure can be designed for using as optical interaction part over a spectral range of interest, and the MMI structure can be used to enable broadband optical coupling from a strip to the slot waveguide for wideband multi-gas on-chip spectroscopic sensing. Reasons for the operating spectral range of the system are discussed.

## 1. Introduction

Photonic integration on silicon (Si) offers a unique opportunity in optical communication, sensing, and spectroscopic applications [1,2,3,4]. For the latter, interaction between evanescent field around Si-based optical waveguide and gas molecules enables the absorption spectrum of sensed gas molecules to be measured in millimeter-sized chips [5,6,7]. With an additional advantage of on-chip integration with Si electronic circuits, Si-based integrated spectroscopic sensing could provide substantial benefits in terms of low cost, small footprint, low power, limited space employment, access to advanced noise reduction, and Si-CMOS high-volume manufacturing [8,9,10,11,12]. Several environmentally-important gas molecules have characteristic absorption lines within the short-wave infrared (~1.4–3 μm), mid-wave infrared (~3–8 μm), or long-wave infrared (~8–14 μm) [13,14]. On the one hand, a number of waveguide platforms have been experimentally or theoretically proposed, such as pedestal Si [15], chalcogenide-based waveguides [16,17], Si-on-sapphire [18], Ge-rich SiGe [19,20,21], and Si-on-Si_3_N_4_ waveguides [22], in order to take advantage of fingerprint absorption in the mid- and long-wave infrared regions. On the other hand, the short-wave infrared regions have also attracted significant attention despite having a weaker overtone absorption [23]. Spectroscopic detection around the ~1.6 μm wavelength range has been promisingly reported when detection at ppmv levels is desirable for a CH_4_ detection using Si waveguide [8], in which conventional optical telecommunication components can be conveniently employed. For the ~2 μm wavelength range, besides its potential for optical communication [23,24], several important gases also have sufficiently strong absorption bands in such wavelength ranges with sensing applications in environmental and process controls such as H_2_O, NH_3_, and CO_2_ [25]. In comparison with the longer wavelength regions, photonic components in the short-wave infrared could be advantageous for the development of low-cost sensors and detectors without cooling [26,27,28]. Recent research investigation has included on-chip room-temperature optical sources and optical detection around ~2 μm wavelength ranges based on heterogeneous integration of III-V semiconductors on silicon such as GeSn [29,30,31], InP [32], strained InGaAs [33,34], or GaSb [23,27]. 

On the other hand, for the optical interaction part with sensed molecules, Si_3_N_4_ on SiO_2_ waveguide platform is one of the Si-compatible platforms suitable for spectroscopic sensing in the short-wave infrared region. Si_3_N_4_ has a relatively large transparent window for the optical wavelength from 0.4 to 5 μm [26,35] and the optical absorption of SiO_2_ increases noticeably at the optical wavelength larger than 3 μm [15,36]. Moreover, due to a moderate refractive index contrast between Si_3_N_4_ waveguide core (n ~ 2) and SiO_2_ cladding (n ~ 1.45), as well as a wider bandgap than that of Si, the Si_3_N_4_/SiO_2_ waveguide platform is also considered to be advantageous in terms of fabrication tolerance and wideband operation, as well as a high optical power handling capability [37]. Additionally, supercontinuum generation has been demonstrated from 1.2 to 3.7 μm spectral range from Si_3_N_4_ waveguides, increasing its suitability for spectroscopic sensing with the promising broadband coherent source in the short-wave infrared region [38].

In this paper, to explore the potential of Si_3_N_4_ on SiO_2_ waveguide platform toward a wideband and simultaneous detection around 2 μm wavelength range, we report on the design of a Si_3_N_4_ on SiO_2_ waveguide architecture that can be simultaneously employed for a wideband on-chip spectroscopic sensing around the 2 μm wavelength region using finite difference eigenmode (FDE) analysis [39] and eigenmode expansion (EME) method (Lumerical Inc.) [40,41]. Firstly, from systematic FDE simulation, we identify a Si_3_N_4_ slot waveguide that is suitable for wideband evanescent sensing around 2 μm optical wavelength. Subsequently, from the EME analysis, a Si_3_N_4_ multi-mode interferometer (MMI) with tapering slot waveguide is interestingly shown to enable simultaneous coupling of light over a spectral range into the identified Si_3_N_4_ slot waveguide for wideband on-chip optical gas sensing, coinciding with the absorption bands of several important gas molecules. From the analysis, we found that over a certain wavelength of interest, a particular slot waveguide structure can be designed for using as optical interaction part, and MMI structure can be employed to enable broadband optical coupling from strip to the slot waveguide for wideband multi-gas on-chip spectroscopic sensing.

## 2. Structure under Investigation: Si_3_N_4_ Multi-Mode Interferometer and Slot Waveguide

**Figure 1:**
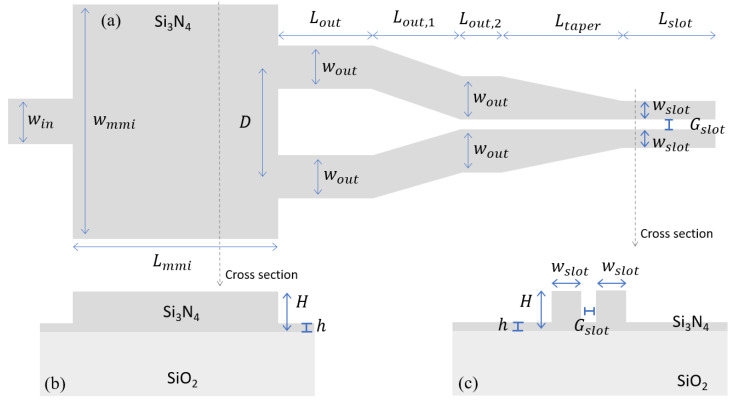
(**a**) Top view and (**b**,**c**) cross-sections of the structure under investigation consist of a Si_3_N_4_ multi-mode interferometer (MMI), tapering slot waveguides, and a slot waveguide with several parameters systematically considered in this work.

As shown in Figure 1, the structure under investigation consists of a Si_3_N_4_ multi-mode interferometer (MMI), tapering slot waveguides, and a slot waveguide. We aim to investigate the possibility to employ such structure for spectroscopic sensing over a wide spectral range around 2 μm wavelength region. The MMI part is used to efficiently couple light from a strip (or rib) waveguide, which can be used to generate or propagate light from the optical source, to a slot waveguide, i.e., the functional region of the sensor. The MMI devices are selected because of its potential for wideband operation and good fabrication tolerance [40]. For increasing optical interaction with the gas molecules, we focus our effort on slot waveguide structures because of its high light–matter interaction potentials [42,43]. It is worth noting that an MMI coupler is required because of the optical mode mismatch between the Gaussian-like mode of the strip waveguide and the non-Gaussian-like mode of the slot one [44]. To thoroughly evaluate the structure for wideband usage, several parameters of the MMI devices with the tapering slot parts and the slot waveguide should be systematically considered, as indicated in Figure 1, including the width of MMI entrance ($w_{in}$) and output ($w_{out}$) waveguides, the width of MMI main waveguide ($w_{mmi}$), the length of MMI main waveguide ($L_{mmi}$), the distance between the two output waveguides (D), the length of different tapering slot parts ($L_{out,1}$, $L_{out,2}$, and $L_{taper}$) between the MMI device and the slot waveguide, the width of a two-side waveguide ($w_{slot}$) and the width of the slot gap ($G_{slot}$) of the slot waveguide, the total height of the Si_3_N_4_ layer (H), and the slab thickness (h). As H is the total Si_3_N_4_ waveguide thickness, if h is not equivalent to zero, the Si_3_N_4_ waveguide will not be etched down to SiO_2_ substrate.

## 3. Slot Waveguides

**Figure 2:**
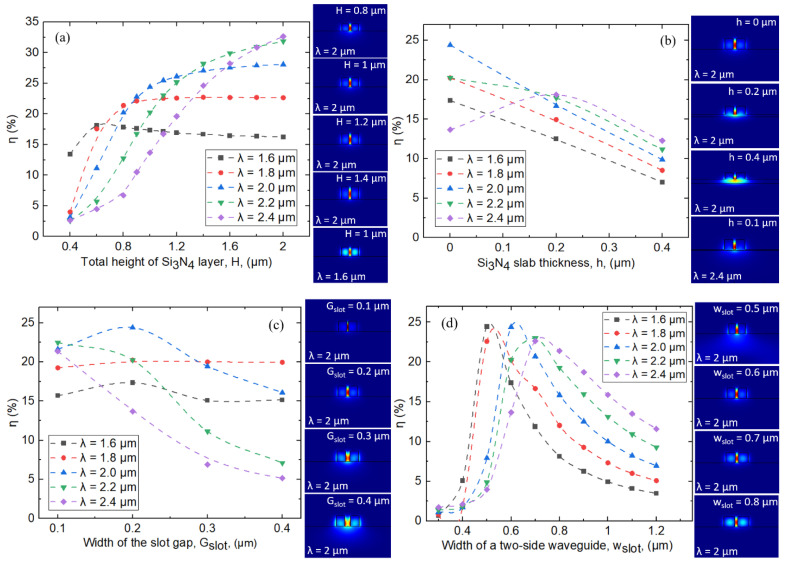
The ratio of the optical power in the air to the total guided optical power, η, in percentage for the slot waveguide with different (**a**) total height of the Si_3_N_4_ layer, H (h, $G_{slot}$, and $w_{slot}$ are kept constant at 0, 0.2, and 0.6 μm), (**b**) slab thickness, h (H, $G_{slot}$, and $w_{slot}$ are kept constant at 1, 0.2, and 0.6 μm), (**c**) width of the slot gap, $G_{slot}$ (H, h, and $w_{slot}$ are kept constant at 1, 0, and 0.6 μm), (**d**) width of a two-side waveguide, $w_{slot}$ (H, h, and $G_{slot}$ are kept constant at 1, 0, and 0.2 μm), at different optical wavelength values from λ = 1.6 to 2.4 µm.

**Figure 3:**
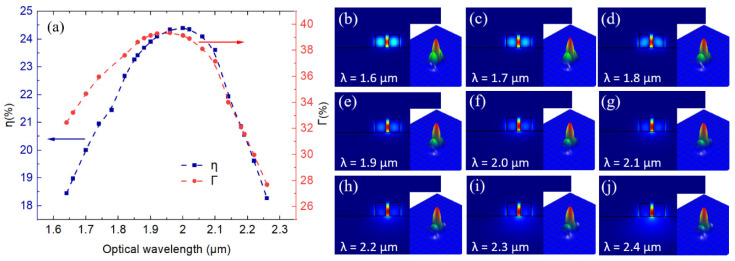
(**a**) Values of η and Γ of the selected Si_3_N_4_ slot waveguide structure with H = 1 μm, h = 0 μm, $G_{slot}$ = 0.2 μm, and $w_{slot}$ = 0.6 μm at different optical wavelength (λ) values. (**b**–**j**) Optical intensity of the quasi transverse-electric (TE) fundamental mode of the Si_3_N_4_ slot waveguide for λ from 1.6 to 2.4 μm. In the inset of Figure 3b–j, 3D mode profiles (Optiwave) of the electric field amplitude are also provided for every corresponding cross-sectional mode profile of optical intensity.

Firstly, we focus on the slot waveguide structure that is suitable for a wideband usage around the 2 μm wavelength range. As the part of optical field which is not confined in the waveguide materials would interact with the gas molecule in the air, the ratio of the optical power in the air to the total guided optical power, $\eta =\iint_{air}^{}P_{z}dxdy/\iint_{total}^{}P_{z}dxdy$, can be used to potentially indicate the strength of light–gas interaction, in which $P_{z}$ is the Poynting vector along the propagation direction [17,39]. Figure 2a–d report η for the slot waveguide with different H, h, $G_{slot}$, $w_{slot}$, and optical wavelength (λ) values from λ = 1.6 to 2.4 µm obtained from FDE analysis. As in Figure 2a, the values of η generally increase with higher values of waveguide height H, while the values of h, $G_{slot}$, and $w_{slot}$ are kept constant at 0, 0.2, and 0.6 μm, respectively. At higher values of H, there is larger area of air gap in the slot waveguide available for optical field as in the inset of Figure 2a for λ = 2 µm. Nevertheless, for the shortest λ of 1.6 µm, the values of η slightly decrease at higher values of slot height H because part of the optical field starts to leak into the two-side waveguides of the slot due to its shorter wavelength values. Therefore, increasing H would instead decrease the fraction of optical power residing in the air, as also shown in the lowest right inset of Figure 2a. To obtain the best overall performance within the spectral range of interest that is compatible with typical fabrication constraints of nowadays high-quality Si_3_N_4_ deposition [45,46], the value of H is restricted to 1 μm in further simulation investigation. For slab thickness h, as in Figure 2b, the values of η generally increase with lower values of h, while the values of H, $G_{slot}$, and $w_{slot}$ are kept constant at 1, 0.2, and 0.6 μm, respectively. This is because more optical field can be resided in the slab part of the waveguide as slab thickness values increase, as in the inset of Figure 2b. However, for λ = 2.4 µm, slab thickness h of 0.1 μm results in the better values of η because the slab layer helps alleviate the leakage of the optical mode into the SiO_2_ substrate for a relatively large wavelength value, as in the lowest right inset. For slot gap $G_{slot}$, as in Figure 2c, the values of η generally decrease with higher values of $G_{slot}$, while the values of H, h, and $w_{slot}$ are kept constant at 1, 0, and 0.6 μm, respectively. $G_{slot}$ of 0.2 μm appears to render the better overall values of η for λ from 1.6 to 2.4 µm. The values of slot gap $G_{slot}$ need to be comparable to the exponential decay length of the fundamental guided mode [47]. For width of a two-side waveguide $w_{slot}$, when the values of H, h, and $G_{slot}$ are respectively kept constant at 1, 0, and 0.2 μm, as in Figure 2d, light at different λ values show different optimized value of $w_{slot}$. Nevertheless, best compromised values of ~0.6 μm could be visibly indicated for $w_{slot}$. A slightly different optimized value of $w_{slot}$ for each optical wavelength is due to the fact that for a given λ value, too small $w_{slot}$ (respectively (resp.) too large $w_{slot}$) would lead to higher leakage into the SiO_2_ substrate (resp. the two-side waveguide part), as illustrated in the inset of Figure 2d for λ of 2 μm. From the optical mode analysis demonstrated in Figure 2a–d in this paper, Si_3_N_4_ slot waveguide structure with H = 1 μm, h = 0 μm, $G_{slot}$ = 0.2 μm, and $w_{slot}$ = 0.6 μm is selected for potential wideband optical sensing considering both the overall η values and practical fabrication constraint of high quality Si_3_N_4_. Besides the η values, $\Gamma =\partial n_{mode}/\partial n_{air\;surrounding}$, when $n_{mode}$ represents effective index of the guided optical mode, can also be used to express the sensitivity of waveguide with respect to the change in the surrounding refractive index, $n_{air\;surrounding}$. Γ was employed to compare different waveguide-based structures for traced gas sensing by spectroscopic absorption [48,49]. Figure 3a summarizes the values of η and Γ of the identified Si_3_N_4_ slot waveguide structure. Respectable values of Γ ~ 30% over a wide spectral range are obtained, comparable to the state-of-the-art devices designed to work at a particular wavelength value of ~1.64 and ~4.24 µm [8,49,50]. To explain the decreasing values of η and Γ at the shorter and longer optical wavelengths, Figure 3b–j show optical intensity of the quasi transverse-electric (TE) fundamental mode of the Si_3_N_4_ slot waveguide from λ = 1.6 to 2.4 μm. At the shorter (resp. longer) optical wavelength regions, optical mode intensity profiles of the first quasi-TE mode become progressively larger in the two-side waveguide parts (resp. SiO_2_ substrate). Therefore, different optical mode profiles at different λ values would play an important role in dictating the spectral range in which a Si_3_N_4_ slot waveguide structure can be employed for optical sensing with good values of η and Γ. In the inset of Figure 3b–j, 3D mode profiles (Optiwave, 32-bit FDTD) of the electric field amplitude are also provided for every corresponding cross-sectional mode profile of optical intensity. The 3D mode profiles of the electric field are excellently agreeable with the cross-sectional one. At the shorter optical wavelengths, optical field of the quasi-TE mode is larger in the two-side waveguide parts. Then, the field is progressively shifted toward the center part of the slot waveguide; nevertheless, from the 3D view it might be more challenging to observe the slight shift of the optical field (the evanescent field) leaked into the substrate at the longer optical wavelengths. As the two data are supportive, we report both cross-sectional and 3D mode profiles. Last, but not least, it is important to note that a vertical slot waveguide will be required to have a smooth etched sidewall to avoid scattering loss due to the sidewall roughness [51]. Fabrication technology already allows smooth etched sidewalls in Si-based slot waveguide recording < 2 dB/cm propagation loss [52,53], and fabrication techniques to achieve micron-scale silicon nitride rib waveguide structure with smooth sidewall are promisingly reported [54].

## 4. Si_3_N_4_ Multi-Mode Interferometer and Slot Waveguide for Wideband On-Chip Optical Gas Sensing

**Figure 4:**
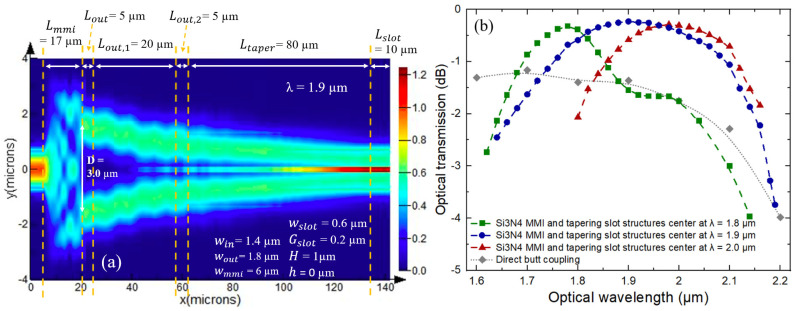
(**a**) Top view optical intensity profile of light propagation from a Si_3_N_4_ strip waveguide via the structure under investigation of a Si_3_N_4_ MMI and tapering slot waveguides to the straight slot waveguide at λ = 1.9 µm, for the structure optimized to have center wavelength at λ ~ 1.9 µm. (**b**) Spectral optical transmission of the MMI and tapering slot structures optimized to have center wavelength at λ ~ 1.8 (green), 1.9 (blue), and 2.0 (red) µm respectively. For the structure with the center wavelength at λ ~ 1.9 µm, an optical transmission loss of 0.23 dB (~5% optical intensity loss) with 3-dB bandwidth (<50% optical intensity loss) of 550 nm from λ = 1.6 to 2.15 µm can be obtained. The gray curve gives spectral optical transmission using direct butt coupling strategy for comparison; obtainable optical transmission decreases by >1 dB over the spectral range of interest.

As aforementioned, we focus on employing Si_3_N_4_ MMI and tapering slot structures for wideband optical coupling from the Si_3_N_4_ strip entrance waveguide into the Si_3_N_4_ slot waveguide (Figure 1a) for spectroscopic sensing using absorption of gas molecules over the spectral range of interest. To identify the most appropriate structure, we proceed to optimize the Si_3_N_4_ MMI and tapering slot parts, and subsequently the spectral optical transmission of each optimized structure is independently investigated to verify its potential for wideband usage with respect to the identified Si_3_N_4_ slot waveguide. For every optimized MMI and tapering slot structure, we start the optimization process of the MMI design by choosing the width of MMI entrance ($w_{in}$) to be 1.4 μm. A relatively large $w_{in}$ is typically preferred in MMI design to reduce refraction caused by a narrower entrance section, and to properly cover the entire wavelength values of interest. Later, we perform EME simulation, a proven method for the design of MMI devices and optical tapers [40], to optimize sequentially the width of MMI main waveguide ($w_{mmi}$), the length of MMI main waveguide ($L_{mmi}$), the distance between the two output waveguides (D), width of MMI output waveguide ($w_{out}$), the length of tapering slot parts ($L_{out,1}$ and $L_{taper}$). The length values of the straight section after the MMI ($L_{out}$) and between the two slot tapers ($L_{out,2}$) are selected to be constant at 5 μm as they are not critical parameters. $w_{mmi}$ needs to be large enough to support enough optical modes for proper interference, $L_{mmi}$ should be consistent with the beat length of the interference, and sufficiently large D would ensure easy fabrication of the trench between the two output waveguides. $L_{out,1}$ and $L_{taper}$ need to be long enough to allow adiabatic transfer of the optical mode from the two MMI output waveguides to the slot waveguide via two tapering slots. Figure 4a reports intensity profile of the optical propagation inside the Si_3_N_4_ multi-mode interferometer and slot waveguide structure at the optical wavelength of 1.9 µm. After optimization, we arrive at $w_{mmi}$ = 6 μm, $L_{mmi}$ = 17 μm, D = 3 μm, $w_{out}$ = 1.8 μm, $L_{out,1}$ = 20 μm, and $L_{taper}$ = 80 μm. It is important to note that the tolerance of an MMI device against wavelength variation depends essentially on the width of the access waveguide, as a wider access waveguide will relax the constrain of wavelength and dimension variation before reaching a certain value of loss penalty [55]. Therefore, using a relatively wide $w_{out}$ of 1.8 μm plays an important role in obtaining a wideband performance from the MMI. A taper structure would later decrease the width to the desired values of 0.6 μm at the slot region. As in Figure 4b (blue curve), the highest optical transmission is obtained around the optical wavelength of 1.9 µm, with an optical transmission loss of 0.23 dB (~5% optical intensity loss), and the structure is found to provide a spectral region with less than 3 dB optical transmission loss (50% optical intensity loss) for λ from 1.6 to 2.15 µm, corresponding to 3 dB optical bandwidth of 550 nm. To explicate, consistent with Figure 3b–j, the optical mode profiles of the targeted slot waveguide become significant in the two-side waveguide parts (resp. SiO_2_ substrate) at the shorter (resp. longer) optical wavelength regions; hence, optical coupling condition via the MMI and tapering slot sections to the slot waveguide will become diverted from the optimized condition at the center wavelength, which is designed to project light into the optical mode at the gap part of the slot waveguide. To verify our explanation, we also investigate spectral optical transmission of the MMI and tapering slot structures optimized to obtain highest optical transmission (optical transmission loss of 0.23 dB) at the lower (resp. higher) optical wavelength value of 1.8 µm (resp. 2 µm). As in Figure 4b, despite having the center wavelength decrease (resp. increase) to 1.8 µm (resp. 2 µm) as in the green (red) curves, optical transmission loss quickly increases to 3 dB at approximately the same optical wavelength values of 1.6 µm (resp. 2.15 µm), affirming that the operating spectral range in which the proposed structure of Figure 1a can be employed for efficient wideband optical coupling is related to optical mode profiles at different optical wavelengths of the selected slot waveguide. Nevertheless, our simulation shows that the MMI and tapering slot structure can be designed to successfully cover all the usable spectral range of the slot waveguide for wideband optical sensing. With respect to the previous works, optical loss of less than 0.45 dB (Over 90% efficiency) from the strip waveguide at the MMI entrance via Si_3_N_4_ MMI and tapering slot structures to the slot waveguide is maintained between 1.82 to 2 µm optical wavelengths (180-nm-wide spectral range), comparable to the previous investigation based on silicon-on-insulator (SOI) waveguide platform between 1.45 and 1.57 μm optical wavelengths (120-nm-wide spectral range) for optical interconnect application [44]. Moreover, optical transmission efficiency from the two waveguides at MMI output to the slot waveguide via the tapering waveguide part is found to be >99%, which is comparable to that reported by [42], in which its architecture on the SOI platform is based on 50/50 power splitter, instead of an MMI, focusing on the optical wavelength of 1550 nm for optical communication. Last, but not least, although direct butt coupling strategy could be used in the SOI platform between 1.45 and 1.65 μm optical wavelengths [56], in our work based on 1-µm-thick Si_3_N_4_ on SiO_2_ waveguide platform, as in Figure 4b (gray curve) we have verified that such approach would decrease obtainable optical transmission by >1 dB around the center wavelength regions, in order to couple optical signal into the Si_3_N_4_ slot waveguide designed for wideband optical sensing between 1.6 and 2.2 µm optical wavelengths.

**Figure 5:**
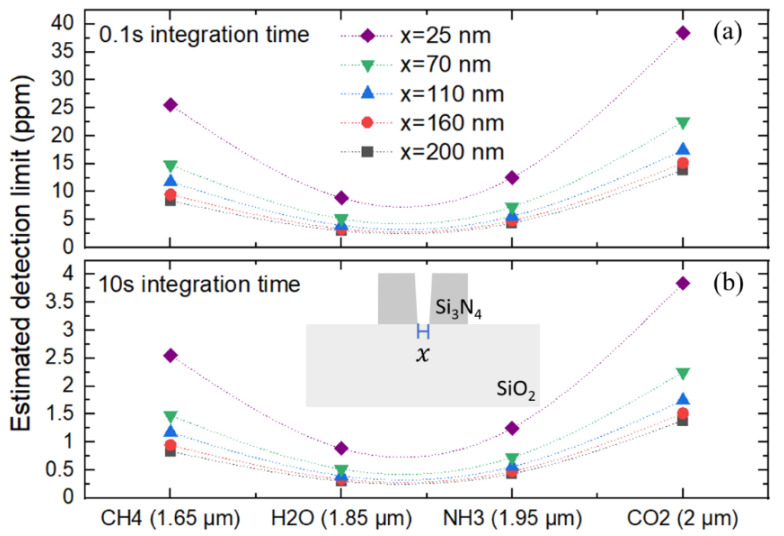
Detection performance with respect to the variation in the slot gap width at the bottom part of the slot (x) from an as-designed slot gap with vertical sidewall (x = 200 nm) to the case that the etching is significantly not completed at the bottom part of the slot (x = 25 nm), taking into account the variation in every section that has 200 nm slot gap including the $L_{out,2}$, $L_{taper}$, and $L_{slot}$ regions. For both (**a**) 0.1 s and (**b**) 10 s integration time, detection performance can be maintained even though the bottom part of the slot is partially etched (x = 160, 110, and 70 nm). Only the significant case of x = 25 nm would result in a considerable deviation of detection performance.

To retrieve the potential of the investigated structure for optical sensing of gas molecules, we evaluated the minimum detectable concentration ($C_{\min}$) of several important gas molecules including methane (CH_4_), water vapor (H_2_O), ammonia (NH_3_), and carbon dioxide (CO_2_), which have absorption lines within the operating bandwidth between 1.6 and 2.2 µm optical wavelengths. $C_{\min}$ of an evanescent field absorption optical sensor can be evaluated by the following expression [39,57,58]:

$$C_{\min}\left(\mathrm{mol}/L\right)=-ln\left[1-\left\{\left(\mathrm{SNR}\cdot \mathrm{NEP}\cdot \sqrt{B}\right)/P_{0}\exp\left(-\alpha L_{\mathrm{opt}}\right)\right\}\right]/{\varepsilon \eta L}_{\mathrm{opt}}$$

where SNR, NEP, and B stand for signal-to-noise ratio, noise equivalent power, and bandwidth of the photodetector employed in the measurement; therefore, these parameters depend critically on the performance of the detection system and operating conditions. $P_{0}$(W) is the optical power arriving at the Si_3_N_4_ slot waveguide, η represents the ratio of the optical power in the air to the total guided optical power as discussed earlier, $\varepsilon \left({\mathrm{Lmol}}^{-1}{\mathrm{cm}}^{-1}\right)$ is molar absorption of gas molecules, $L_{\mathrm{opt}\;}$(cm) is the optimal waveguide length, and $\alpha \left({\mathrm{cm}}^{-1}\right)$ is the waveguide intrinsic optical loss. We used relatively typical values of SNR = 10, NEP = 5 × 10^−12^ W/√Hz, and B = 5 kHz (0.1 ms integration time), 5 Hz (0.1 s integration time), or 0.05 Hz (10 s integration time) [4,6,18,57,58]. The value of NEP was estimated from available commercial photodetector within the optical wavelength of interest (UPD-3N(5N)-IR2-P, alphalas.com), and being conservative with respect to the number used in previous works at the longer wavelength values [18,58,59]. It is worth noting that around the 2 µm optical wavelengths, operation at room temperature is possible, and thermal noise dominates [18]. The integration time, t, can be related to the bandwidth (BW) of the photodetector as t=1/(2·BW) [60]. Assuming optical power of 1 mW at the entrance of the MMI, $P_{0}$ arriving at the Si_3_N_4_ slot waveguide for each optical wavelength is then 1 mW corrected by the optical transmission spectra of the MMI and tapering slot structures obtained in Figure 4b (blue curve). α is estimated to be 2 dB/cm, which results in $L_{\mathrm{opt}\;}$ of ~2 cm [4]. Si_3_N_4_ was projected to be a low-loss platform at the wavelength of interest [3], and a low-loss Si_3_N_4_ on oxide waveguide was demonstrated up to the optical wavelength of 2.6 um (0.6 dB/cm) [35,61,62]. Therefore, an estimation of 2 dB/cm propagation loss should be considered reasonably conservative. Molar absorption values, ε, of each gas molecule are obtained from the HITRAN database and summarized in Table 1 at different optical wavelengths [63]. η values of the slot waveguide are summarized in Figure 3a. Although, as expected, the molar absorption values in this short-wave infrared region are generally lower than those in the mid-wave infrared region [39], minimum detectable concentration at ppm levels can be attained with reasonable values of integration time. These estimated detection limit values are lower than the occupational exposure limit of 1000 ppm, 25 ppm, and 5000 ppm for CH_4_, NH_3_, and CO_2_ recommended by the international environmental standards, respectively [64]. For water vapor, the resolution limit of a few ppm already makes it compatible with the moisture level required for industrial processes [65]. Moreover, the estimated detection limit values CH_4_, NH_3_, and CO_2_ are compatible with the concentration needed to be regulated in pig houses of ~24, ~12, and ~500 ppm, respectively, as measured by a free space configuration [66], affirming potential applications of the investigated structure. It should be noted that although the detection limit of free-space configuration will typically be lower than the waveguide one thanks to the much longer interaction length, the figure of merit when considering both the detection limit and device’s compact size can be advantageous for waveguide configuration [59]. In addition, the definition of a tight slot gap should be one of the most critical steps that could affect the projected detection performance, despite significant development on etching technology to obtain vertical and smooth sidewall in Si-based devices [52,53,54]. Therefore, we investigate the detection performance with respect to the variation in the value of gap width at the bottom part of the slot (x) from an as-designed slot gap with vertical sidewall (x = 200 nm) to the case that the etching is significantly not completed at the bottom part of the slot (x = 25 nm), taking into account the variation in every section that has 200 nm slot gap including the $L_{out,2}$, $L_{taper}$, and $L_{slot}$ regions. As in Figure 5a,b, detection performance can be maintained even though the bottom part of the slot is partially etched (x = 160, 110, and 70 nm.) Only the significant case of x = 25 nm would result in a considerable deviation of detection performance.

## 5. Conclusions

The potential of Si_3_N_4_ on SiO_2_ waveguide platform toward a wideband spectroscopic detection around the optical wavelength of 2 μm is investigated. A Si_3_N_4_ slot waveguide with capability to facilitate wideband on-chip spectroscopic sensing around 2 μm is identified by systematic optical mode analysis, and from EME investigation, a Si_3_N_4_ MMI with tapering slot waveguides can be employed for efficient optical coupling from a strip waveguide to the identified slot waveguide over its entire usable spectral range from 1.6 to 2.2 µm optical wavelengths. Optical mode profiles at different optical wavelengths of the slot waveguide would play an important role in determining the useful spectral range of the system for optical sensing based on optical absorption of the evanescent field. From the analysis, the detection limit of the investigated structure is compatible with the requirements for environmental and agricultural usages. As on-chip broadband light source and spectrometer systems [37,38] are already investigated in Si_3_N_4_ systems, this investigation provides further information toward the development of Si_3_N_4_-based on-chip spectroscopic sensing.

## Figures and Tables

**Table 1:** Molar gas absorption data of each gas molecule with absorption lines between 1.6 and 2.2 optical wavelengths [63] and corresponding projected detection resolution.

Gas Molecules	Optical Wavelength (µm)	Molar Absorption (Lmol^−1^cm^−1^)	Estimated Detection Limit (ppm)		
			Integration Time (second)		
			0.1 ms	0.1 s	10 s
CH_4_	1.65	~3.8	262	8.38	0.838
H_2_O	1.85	~5.2	94.3	3.01	0.301
NH_3_	1.95	~3.4	136	4.37	0.437
CO_2_	2	~1.1	437	13.9	1.39

## Data Availability

Not applicable.
